# Resistant Thyrotoxicosis due to Graves' Disease in Pregnancy: Case Report and Review of the Literature

**DOI:** 10.7759/cureus.3232

**Published:** 2018-08-30

**Authors:** Anastasia Linardi, Ekaterini Michou, Ioannis Ilias, Foteini Petychaki, Ioannis Kakoulidis, Athina Pappa, Eftychia Koukkou

**Affiliations:** 1 Department of Endocrinology, Diabetes and Metabolism, E. Venizelou Hospital, Athens, GRC; 2 Department of Endocrinology, Diabetes and Metabolism, E. Venizelou Hospital, Athens , GRC

**Keywords:** female, humans, hyperthyroidism, pregnancy, complications, thyroid diseases

## Abstract

The effective management of Graves' disease (GD) during pregnancy is crucial for maternal and neonatal well-being. Conventional treatment of GD during pregnancy includes antithyroid drugs (ATDs) and surgery, ideally during the second trimester. We report a 27-year-old woman with GD and we present the course of GD during her three consecutive pregnancies. During the first pregnancy, thyrotoxicosis was successfully treated with low doses of antithyroid drugs; in the second pregnancy, thyrotoxicosis was only controlled at the third trimester; while in the third pregnancy, our patient presented with treatment-resistant thyrotoxicosis, which was finally managed with corticosteroids in adjunction with ATDs. Although hyperthyroid, the patient maintained her fertility. Resistance to ATD is a rare condition and in our case was adequately controlled with corticosteroids.

## Introduction

Graves’ disease (GD) is an autoimmune disorder involving the thyroid gland, typically characterized by the presence of circulating autoantibodies that bind to and stimulate the thyrotropin receptor (TRAbs), resulting in hyperthyroidism and diffuse goiter [1]. GD is a rare condition during pregnancy, affecting 0.2% of all pregnant women [1].

The effective management of GD during pregnancy is challenging for the clinician [2]. During the first trimester, due to the stimulation of the thyroid gland by human chorionic gonadotrophin (hCG) or an elevation in thyrotropin/thyroid-stimulating hormone (TSH) receptor antibodies (TRAb) levels, transient worsening of the GD symptoms may be observed. As pregnancy progresses, the concomitant immunosuppression usually leads to improvement in symptoms. However, postpartum, as immune system function rebounds, GD exacerbates. Poor control of thyrotoxicosis is associated with maternal, fetal, and neonatal complications [1, 3]. Maternal complications include left ventricular dysfunction and thyroid storm, while obstetric complications include miscarriage, prematurity, preeclampsia, placental abruption, postpartum haemorrhage, and stillbirth. Moreover, there is an increased risk of neonatal thyroid dysfunction and of low birth weight.

Treatment with antithyroid drugs (ATDs) is related to adverse effects such as teratogenicity, hepatotoxicity, and agranulocytosis [1]. Clinicians should administer adequate ATDs to sufficiently control maternal hyperthyroidism and at the same time avoid overtreatment and induction of fetal and neonatal hypothyroidism [1, 2]. On the other hand, the presence of persistently elevated TRAb levels during pregnancy is prognostic of fetal hyperthyroidism. In the general population, ATDs are usually chosen for the management of GD, leading to remission in around 50% of patients after 12-18 months of treatment [4]. The ATDs are thionamides (propylthiouracil-PTU, methimazole-MMI, and carbimazole-CBZ), which inhibit thyroid hormone synthesis. In the presence of a large goiter, in case of intolerance to ATDs or of disease recurrence, surgery is an alternative treatment. Radioidine therapy, which is the preferred option for GD in the United States, is contraindicated in pregnancy.

We describe the course of thyrotoxicosis during three consecutive spontaneous pregnancies of a 27-year-old (at first presentation) woman with a known history of GD. During the third pregnancy, the patient developed thyrotoxicosis resistant to ATD. Finally, the disease was adequately controlled with corticosteroids.

## Case presentation

**Figure 1:**
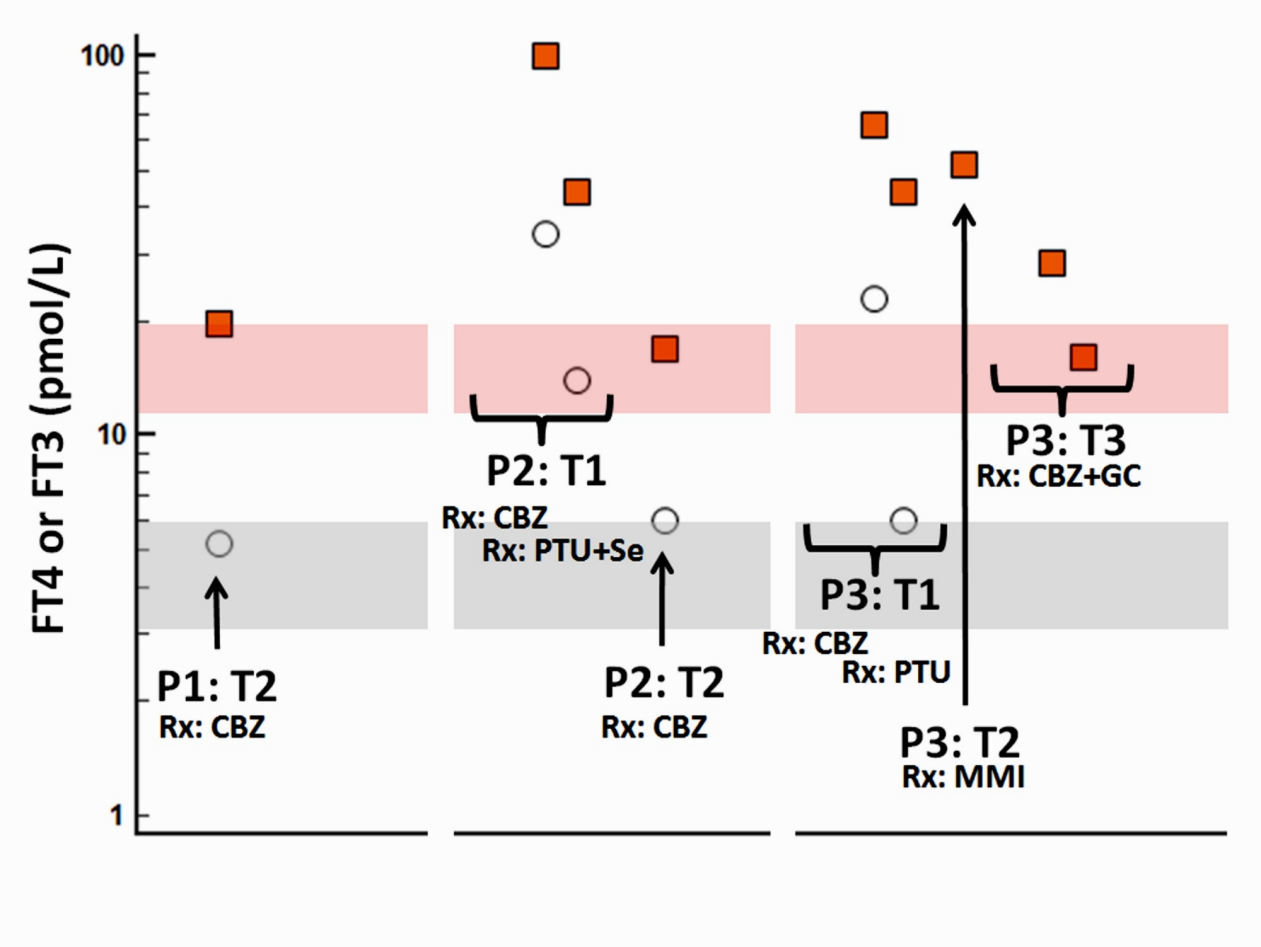
Schematic representation of the patient’s free thyroxine (FT4) and free triiodothyronine (FT3) levels in her three consecutive pregnancies vis-à-vis treatment Red squares indicate FT4 and open circles indicate FT3 levels; red shaded areas denote the normal FT4 range and grey shaded areas denote the normal FT3 range; P1: T2: second trimester of the first pregnancy; P2: T1: first trimester of the second pregnancy; P2: T2: second trimester of the second pregnancy; P3: T1: first trimester of the third pregnancy; P3: T2: second trimester of the third pregnancy; P3: T3: third trimester of the third pregnancy; Rx: treatment; CBZ: carbimazole; PTU: propylthiouracil; Se: selenium; MMI: methimazole; GC: corticosteroids.

A 27-year-old woman with known history of GD from seven years presented at the 21st week of her first spontaneous pregnancy. She was on long-term CBZ (10 mg daily) treatment and was clinically euthyroid. Thyroid function tests were compatible with subclinical hyperthyroidism, with free thyroxine (FT4) of 19.6 pmol/L (normal range: 12-22), free triiodothyronine (FT3) of 5.2 pmol/L (normal range: 3.1-6.8), and TSH of 0.05 mIU/L (normal range: 0.25-4.5) (Figure 1).

The TRAbs assay was negative. The patient remained euthyroid and the TRAbs remained negative throughout pregnancy. She delivered normally without complications (the neonate’s birth weight was 2500 gr). Postpartum the patient continued CBZ 10 mg daily. 

Two months later she was pregnant again, having also symptoms of thyrotoxicosis: palpitations, heat intolerance, sleep disturbances, as well as bilateral exophthalmos. On clinical examination, she had sinus tachycardia with a heart rate of 115/min, diffuse goiter with a bruit, and fine tremor in her hands. Thyroid function tests revealed a fivefold rise of FT4 levels (FT4: 100 pmol/L, normal range: 12-22), a sixfold rise of FT3 levels (FT3: 34.6 pmol/L, normal range: 3.1-6.8) (Figure 1), and a suppression of TSH levels (TSH: <0.01 mIU/L, normal range: 0.25-4.5). The TRAb levels were elevated as well (TRAbs: 16 U/L, normal range: <1.75). Treatment was changed to PTU (300 mg daily) and selenium (200 mg daily) with only partial response, as the FT4 and FT3 levels decreased to twice normal. During the second trimester, PTU was switched to 20 mg of CBZ daily; thyrotoxicosis was adequately controlled in the 36th week of pregnancy, with normal FT4 and FT3 levels and suppressed TSH. The patient delivered with a selective caesarean section (CS) at the 38th week of pregnancy. The TRAb levels remained positive throughout the second pregnancy (TRAbs at 34th week of pregnancy: 2.03 U/L, normal range <1.75). Neither the mother nor the neonate had complications (neonate birth weight: 2890 gr). Postpartum, the patient was advised to increase the dose of CBZ to 30 mg daily and thyroidectomy was scheduled.

The scheduled thyroidectomy was not performed, as within a few months the patient was pregnant for the third time. She presented with more accentuated symptoms of palpitations, tremor, heat intolerance, irritability, bilateral exophthalmos, and diffuse goiter. Thyroid function tests revealed severe thyrotoxicosis with TSH < 0.005 mIU/mL, (normal range: 0.25-4.5), FT4: 66.25 pmol/L, (normal range: 12-22), and FT3: 23.04 pmol/L, (normal range: 3.1-6.8). During the third pregnancy, the patient developed resistance to ATDs. More in detail, during the first trimester PTU was initiated. Her FT4 failed to normalize despite high doses of PTU (400 mg daily) (Figure 1). In the second trimester, a switch to maximal doses of MMI (60 mg daily) had a minimal benefit on FT4, which reached 43 pmol/L (normal range: 12-22); it was discontinued and CBZ was started at maximal doses (45-60 mg daily) with no response (FT4: 52.4 pmol/L). Although the patient’s compliance was questioned, careful in-hospital observation and inspection did not disclose any infractions. Her TRAb levels remained remarkably elevated throughout pregnancy. In the 21st week of pregnancy they had reached a peak of 27.7 U/L (normal range <1.75); the elevated TRAb persisted, since in the 30th week a value of 7.48 U/L was found.

Given the poor control of maternal thyrotoxicosis, the high doses of ATDs, and the elevated TRAb levels during pregnancy, thorough fetal surveillance was performed to minimize possible complications. Serial fetal ultrasounds were performed for the assessment of fetal viability, fetal growth, amniotic fluid volume, fetal anatomy, and detection of malformations. Moreover, a fetal cardiac ultrasound in the 30th week of pregnancy excluded fetal tachycardia and signs of congestive heart failure. Propranolol was also administered to control the symptoms of thyrotoxicosis. In the 32nd week of pregnancy, the patient was hospitalized and, given the increased risk of preterm delivery, antenatal corticosteroids were administered for fetal lung maturation [5].

Since a remarkable improvement in thyroid function was observed within a few days of starting corticosteroids, with FT4 levels reaching 29.3 pmol/L (normal range: 12-22), corticosteroid administration was kept along with CBZ [6, 7]. A 30 mg daily dose of prednisolone led to normalisation of FT4 levels within one week (FT4: 15.9 pmol/L) (Figure 1).

The patient underwent CS at the 37th week without complications. The neonate weighed 2280 gr and was hospitalized for three months because of staphylococcal encephalitis unrelated to the mother’s GD. Postpartum, treatment included 20 mg of prednisolone in adjunction with 15 mg of CBZ per day. Surgical treatment was planned. Thyroidectomy was performed two months later without complications. Prednisolone was gradually withdrawn; the patient is currently treated with thyroxine and is clinically well.

## Discussion

Hyperthyroidism is generally associated with infertility, though there is limited and sometimes conflicting evidence of this association [8]. Surprisingly, our patient had never had menstrual disturbances and while thyrotoxic, she had three spontaneous consecutive pregnancies. Prospective studies have demonstrated that hyperthyroidism is found in 2.3% of infertile women, while retrospective studies show that 5.8% of hyperthyroid women have infertility [8].

Menstrual disturbances, mainly amenorrhea, oligomenorrhea, and hypomenorrhea have been described in 21% to 65% of women with hyperthyroidism [9]. According to endometrial biopsies, most hyperthyroid women, remain ovulatory. Animal studies have shown that ovaries of hyperthyroid rats have more atretic follicles compared to ovaries of euthyroid rats. Hyperthyroidism is characterized by hormonal alterations that may partially explain infertility in women [8]. Plasma estrogen levels are two to threefold higher than in normal women in all phases of the menstrual cycle. Moreover, there is an increase in sex hormone-binding globulin (SHBG) [9]. Testosterone and androstenedione levels are also increased in hyperthyroid women, and their conversion rates to estradiol and estrone, respectively, are elevated [9]. Hyperthyroxinemia produces an augmented responsiveness of gonadotropins to gonadotropin-releasing hormone (GnRH), and as a result, mean luteinizing hormone (LH) concentration is found increased in all phases of the menstrual cycle. Hyperthyroidism may influence fertility not only through hormonal changes but also through thyroid autoimmunity (TAI). It is proposed that the abnormal autoimmune background which coexists with TAI has a direct negative impact on female fertility. Moreover, TAI is associated with other causes of infertility, such as premature ovarian failure, endometriosis, and polycystic ovarian syndrome. In the follicular fluid there are thyroid autoantibodies which possibly are cytotoxic to oocytes [10]. Thyroid hormone receptors (THR) and TSH receptors (TSHR) are found in ovarian granulosa and stromal cells [8] and are expressed in the human endometrium throughout the menstrual cycle. Thyroid hormones (TH) down-regulate aromatase activity in granulosa cells, improve granulosa cells proliferation, inhibit granulosa cell apoptosis and play an important role in the human endometrium, especially during implantation. Furthermore, TH may have an impact on uterine oxidative stress, thus affecting female fertility. In addition, leptin, which is increased in hyperthyroidism, influences fertility, mainly during implantation.

Thyrotoxicosis during pregnancy is associated with adverse events for the mother as well as for the fetus. It is therefore critical to achieve rapid and adequate control of the disease. Medical therapy with ATDs remain the mainstay of treatment for pregnant women with GD [3]. The majority of patients with GD respond to treatment with ATDs. However, sporadic cases of resistant thyrotoxicosis are reported. Our patient is unique because she developed resistance to all ATDs during pregnancy. Possible mechanisms responsible for resistance to ATDs include drug malabsorption, antidrug antibodies, rapid drug metabolism, and impaired intrathyroidal drug accumulation or action [11]. Among patients with poor response to ATDs, noncompliance is the most likely culprit [12]. In our patient, the enforced strict observation should have eliminated the possibility of noncompliance to therapy. Malabsorption was ruled out through careful history taking and physical examination. The hypothesis of drug resistance can be confirmed by the measurement of drug levels and antidrug antibodies [6, 13], methods that are not easily available and which were not performed. A perchlorate discharge test, four hours after drug intake under medical supervision is an alternative test to confirm resistance to ATD. A negative test indicates an inadequate blockade of iodide organification and thus possible drug resistance, while a positive test indicates some degree of iodide organification and implies lack of compliance [12, 13]. In this case, if available, perchlorate could only have been given when our patient was not pregnant. Iodine contamination could also have been ruled out by measuring urinary iodine excretion [13]. Iodine changes the response of the thyroid to ATDs, by altering intrathyroidal metabolism of the drugs, by increasing TH stores, and by delaying the clinical therapeutic effect [14].

Resistant thyrotoxicosis is managed by definitive treatment options: surgery or radioactive iodine ablation. Before these interventions, patients should be euthyroid to decrease perioperative adverse events, such as precipitation of a thyroid storm. To achieve euthyroid state rapidly, different approaches have been used. Iopanoic acid, an oral cholecystographic agent has been used for rapid preoperative preparation of uncontrolled, severe thyrotoxicosis due to GD refractory to propranolol and ATD in a 14-year-old boy [15]. Iopanoic acid may improve hyperthyroidism by several mechanisms: it inhibits the conversion of T4 to T3, reduces tissue uptake of TH, decreases TH synthesis, decreases thyroidal response to TSH, and decreases release of TH from the thyroid gland [15]. Yet, long term treatment with iopanoic acid is not feasible because of the relapse of thyrotoxicosis due to an escape phenomenon [15]. Cholestyramine, a bile acid sequestrant, when added to ATDs, produces a rapid decline in serum TH levels [16]. Lithium, used for bipolar affective disorder, increases retention of radioactive iodine in the thyroid gland and has been successfully used as an adjunct to radioactive iodine in such cases [6]. Corticosteroids are generally used in thyroid storm [7]. Sporadically, they have been used preoperatively or before administration of radioiodine to achieve a euthyroid state in patients with resistant thyrotoxicosis [6, 7]. Jude et al. have reported two cases of thyrotoxicosis resistant to maximal doses of CBZ [7]. Prednisolone was administered in addition to CBZ with dramatic response before treatment with radioiodine [7]. The authors have proposed two possible mechanisms of action for the rapid normalization of TH concentrations: inhibition of peripheral conversion of thyroxine (T4) to triiodothyronine (T3) and suppression of the immune response leading to decreased stimulation of the thyroid gland [7, 17]. Saleem et al., reported the case of a 50-year-old woman with resistant thyrotoxicosis who became euthyroid with the administration of prednisolone, lithium, and ATDs before radioactive iodine ablation [6]. Min et al. reported an extremely rare case of therapeutic plasmapheresis in a patient with GD before radioactive iodine treatment [18]. The ATDs that were used initially were discontinued, due to compromised liver function, and replaced by Lugol’s solution. Subsequently, due to an escape phenomenon (to Lugol’s solution) therapeutic plasmapheresis was performed and managed to control the thyrotoxicosis [18]. A short course of Lugol solution can be given with propranolol to achieve euthyroidism before total thyroidectomy. However, the use of iodides during pregnancy is usually contraindicated because of their association with neonatal goiter and hypothyroidism.

We have to point that propranolol was also administered to control the symptoms of thyrotoxicosis in our patient, even though it is associated with intrauterine growth restriction, fetal hypoglycaemia, and bradycardia [19]. This was done to control her symptoms and was given with the patient’s consent after discussion. From the aforementioned treatment options, only the addition of corticosteroids to ATDs is compatible with pregnancy. Dexamethasone should be avoided, as it is not inactivated by placental 11β-hydroxysteroid dehydrogenase type 2 and thus crosses the placenta to the fetus [20]. We therefore decided to administer 30 mg of prednisolone with maximal doses of CBZ with rapid remission of thyrotoxicosis. In such high doses, prednisolone can saturate the placental enzymes and, as a result, large amounts of corticosteroids can cross the placental barrier causing significant suppression of the fetal adrenal glands [20]; notably, fetal adrenal suppression develops approximately within 14 days after maternal steroid use. No such suppression was noted in the neonate, despite prolonged hospitalization for unrelated causes.

## Conclusions

We described a rare case of apparent resistance to ATDs during pregnancy. Given the fact that poorly controlled thyrotoxicosis is related to severe maternal/fetal complications, it is crucial to achieve euthyroidism rapidly. In such cases, especially around the time of parturition, a short-term course of corticosteroids should be considered as adjunctive therapy, as it can lead to rapid clinical and biochemical control of thyrotoxicosis.
